# Symptom control and health‐related quality of life in allergic rhinitis with and without comorbid asthma: A multicentre European study

**DOI:** 10.1002/clt2.12209

**Published:** 2023-02-02

**Authors:** Subhabrata Moitra, Marzia Simoni, Sandra Baldacci, Sara Maio, Anna Angino, Patrizia Silvi, Giovanni Viegi, Stefania La Grutta, Franco Ruggiero, Gianni Bedini, Francesca Natali, Lorenzo Cecchi, Uwe Berger, Maria Prentovic, Amir Gamil, Nour Baïz, Michel Thibaudon, Samuel Monnier, Davide Caimmi, Luciana K. Tanno, Pascal Demoly, Simone Orlandini, Isabella Annesi‐Maesano

**Affiliations:** ^1^ Division of Pulmonary Medicine & Alberta Respiratory Centre Department of Medicine University of Alberta Edmonton Alberta Canada; ^2^ Pulmonary Environmental Epidemiology Unit CNR Institute of Clinical Physiology (IFC) Pisa Italy; ^3^ CNR Institute of Translational Pharmacology (IFT) Palermo Italy; ^4^ Department of Biology University of Pisa Pisa Italy; ^5^ Department of Agrifood Production and Environmental Sciences University of Florence Florence Italy; ^6^ Centre of Bioclimatology University of Florence Florence Italy; ^7^ Research Unit Aerobiology and Pollen Information Department of Oto‐Rhino‐Laryngology Medical University of Vienna Vienna Austria; ^8^ Institut Desbrest of Epidemiology and Santé Publique INSERM & Montpellier University Montpellier France; ^9^ Reseau National de Surveillance Aerobiologique (RNSA) Brussieu France

**Keywords:** allergy treatment, CARAT, food allergy, pollen, RHINASTHMA, rhinitis

## Abstract

**Background:**

Allergic rhinitis (AR) is a major non‐communicable disease that affects the health‐related quality of life (HRQoL) of patients. However, data on HRQoL and symptom control in AR patients with comorbid asthma (AR + asthma) are lacking.

**Methods:**

In this multicentre, cross‐sectional study, patients with AR were screened and administered questionnaires of demographic characteristics and health conditions (symptoms/diagnosis of AR and asthma, disease severity level, and allergic conditions). HRQoL was assessed using a modified version of the RHINASTHMA questionnaire (30, ‘not at all bothered’ ‐ 150 ‘very much bothered’) and symptom control was evaluated by a modified version of the Control of Allergic Rhinitis/Asthma Test (CARAT) (0, ‘no control’ ‐ 30, ‘very high control’).

**Results:**

Out of 643 patients with AR, 500 (78%) had asthma as a comorbidity, and 54% had moderate‐severe intermittent AR, followed by moderate‐severe persistent AR (34%). Compared to the patients with AR alone, patients with AR + asthma had significantly higher RHINASTHMA (e.g., median RHINASTHMA‐total score 48.5 vs. 84, respectively) and a significantly lower CARAT score (median CARAT‐total score 23 vs. 16.5, respectively). Upon stratifying asthma based on severity, AR patients with severe persistent asthma had worse HRQoL and control than those with mild persistent asthma. The association was significantly higher among non‐obese participants compared to obese ones, with RHINASTHMA‐upper symptoms score but not with CARAT.

**Conclusions:**

Our observation of poorer HRQoL and symptoms control in AR patients with comorbid asthma supports the importance of a comprehensive approach for the management of AR in case of a comorbid allergic condition.

## 1 INTRODUCTION

Allergic rhinitis (AR) is a type‐2 chronic inflammatory disease affecting the nasal mucosa and characterized by nasal symptoms such as sneezing, rhinorrhoea (nasal discharge), pruritus, and nasal congestion.^1^, ^2^, ^3^ It is one of the most common non‐communicable chronic diseases in the world, affecting over 400 million people of all ages, particularly the paediatric population.^1^, ^2^, ^3^, ^4^, ^5^, ^6^ While the prevalence of physician‐diagnosed AR in the United States has been observed as high as 15% and 30%, based on self‐reported nasal symptoms,^7^, ^8^ the prevalence was as high as up to 50% in many European countries.^9^ According to the Allergic Rhinitis and its Impact on Asthma (ARIA) and the Global Alliance against Chronic Respiratory Diseases (GARD) statements, severe, refractory, or mixed forms of AR are significantly increasing across the globe and have contributed substantially to the socio‐economic burden of the disease.^10^, ^11^, ^12^

Allergic rhinitis often coexists with other conditions, such as atopic dermatitis, rhinosinusitis, rhino‐conjunctivitis, and particularly asthma – a coherent feature often referred to as ‘the atopic March’ due to common systemic inflammatory processes.^2^, ^4^ 40%–50% of patients with AR also have asthma whereas the prevalence of AR as a comorbidity in asthmatic patients is even higher, that is, 70%–90%.^13^ Several reports described that the patients suffering from AR show a poorer quality of life (QoL), being affected by impaired sleep patterns, increased amount of fatigue, depression, risk of driving accident, and altered physical and social functions.^8^, ^14^, ^15^, ^16^ Often, a poor perception of AR symptoms is associated with poor control of AR.^17^ However, studies assessing health‐related quality of life (HRQoL) and symptoms control in AR patients with concomitant asthma are lacking.

The Aerobiological Information Systems and allergic respiratory disease management (AIS Life +) study focused on this aspect, by using specifically designed and validated questionnaires on QoL and control for AR with comorbid asthma. In this multicentre study, using validated questionnaires, we aimed to assess the differences in symptom control and HRQoL between AR patients with or without comorbid asthma.

## 2 METHODS

### 2.1 Study design and participants

In the international multi‐centre (Austria, France, and Italy) cross‐sectional AIS Life + study, conducted between 2013 and 2014, we enrolled participants suffering from nasal allergy. A convenient sample of individuals with an active condition of pollen‐induced AR was selected from pre‐existing epidemiological study databases or through web advertisement (Pisa, Italy), clinics of general practitioners (Paris, France) or public health databases and pulmonary clinics (Vienna, Austria) and invited to participate in this epidemiological survey. All potential participants were administered a screening questionnaire through a telephone interview to check whether they were eligible for the study. We included participants who: (1) were adults (≥18 years of either sex); (2) reported a physician‐diagnosed pollen‐induced allergic rhinitis/hay fever or nasal symptoms or positive clinical tests to pollen (skin prick tests or specific immunoglobulin E [IgE]) in the last 12 months; (3) spent most of the week (at least 5 days/week) living, studying, or working in the areas where this study was conducted; and (4) were not treated with allergen immunotherapy over the previous 6 years.

The study was approved by the ethics committees of the participating centres in Italy (Ethics Committee of University‐Hospital of Pisa; Protocol No. 14248) and in Austria (Berlin Charite University Ethics: EA1/119/12), and signed informed consents were obtained from all the participants before recruitment. In France, the approval by an external ethic committee was declared as not applicable at that time: instead, the study was approved by the hospital ethics committee, by the National Committee for Information Management on Medical Research (*Comite´ Consultatif sur le Traitement de l’Information en Matière de Recherche dans le domaine de la sante´*) and by the National Commission on Informatics and Health (CNIL, *Commission Nationale Informatique et Liberté*). In France, the CNIL approved in 2016 that all data acquired before 2016, without the previous need for authorization from an Ethics Committee, could still be utilized. In any case, patients were seen in the frame of routine care (“*soins courants*”). The AIS study was conducted according to the Declaration of Helsinki and reported as per the Strengthening the Reporting of Observational Studies in Epidemiology (STROBE) guidelines.^18^

### 2.2 Instruments and variables

A standardized health questionnaire was administered to all eligible individuals in order to obtain information on their demographic characteristics (age, gender, body mass index [BMI], level of education), and potential risk factors (smoking status, exposure to second‐hand smoke, and drug consumption), on symptoms/diagnosis of AR and asthma, disease severity level, as well as on previous clinical tests (spirometry, skin prick test and serum level of IgE).

The HRQoL of the participants was assessed using a modified version of the validated RHINASTHMA questionnaire (the higher the score, the lower the HRQoL), that is, the only available instrument that allows evaluating the concomitant impact of AR and asthma on HRQoL.^19^ This 30‐item questionnaire provides individual scores for upper airways (RHIN‐Upper), lower airways (RHIN‐Lower), respiratory allergy impact (RHIN‐RAI), and a composite/total score (RHIN‐Total or the Global Summary [RHIN‐GS] score, which indicates the overall impact of the disease). The details of the instrument and the scoring system can be found elsewhere.^19^ Patients used a five‐point Likert scale (‘not at all’, ‘a little’, ‘fairly’, ‘much’, ‘very much’) to indicate the extent to which they were bothered by each AR and asthma during the year preceding the completion of the questionnaire. These responses are then converted into scores, from 0 to 100, with larger scores corresponding to worse HRQoL. A RHINASTHMA‐GS score from 0 to 20, indicating minimal or absent disease impact on patient life, was considered reflective of optimal HRQoL. For this analysis, we calculated the scores (ranging between 30 = possible total best score and 150 = possible total worst score) as described previously.^20^ For this modified version, we used an English‐back translation of the instrument as the questionnaire was not available or validated in local languages.

The control of AR and asthma was evaluated by a modified version of the Control of Allergic Rhinitis/Asthma Test (CARAT) (the higher the score, the higher the disease control).^21^, ^22^ Control of Allergic Rhinitis/Asthma Test is a 10‐item questionnaire containing information about the frequency of symptoms, sleep impairment, activities limitation, and need for more medication: the response options for all the questions follow a 4‐point Likert scale (range 0–3). The range of CARAT score is 0–30, 0 being the complete absence of control and can address upper airway symptoms (CARAT‐Upper), lower airway symptoms (CARAT‐Lower) in addition to the summary score (CARAT‐Total) with the minimal clinically important difference of 3.5.^23^ The Global Initiative for Asthma (GINA) classification 2017^24^ and ARIA (2008)^6^ were used to classify asthma according to its severity. Control of Allergic Rhinitis/Asthma Test is available in several languages including those of the participating countries.

#### 2.2.1 Statistical analyses

Data were described as frequency (%), mean (standard deviation [SD]), or median (interquartile range [IQR]) for categorical, continuous, and ordinal variables, respectively. To test the association between QoL and control (RHINASTHMA and CARAT – Total and subdomains) scores, and AR + asthma (independent variable), we first used a bivariate analysis using Wilcoxon rank‐sum test. Then, we constructed univariable (unadjusted) and multivariable (adjusted) regression models among the independent variable and HRQoL and control scores using a mixed effect Poisson regression model. As potential confounders, we tested fixed factors (age, sex, BMI, smoking status, exposure to smoke, education, ARIA grade, sensitivity to allergens, and drugs taken in the last 12 months) and a random factor (the country). To include confounders in the regression models, we used a priori evidence criteria, that is, covariates were considered as confounders if were found consistent in previous literature. However, confounders were retained in the model if they modified the estimates of the remaining variables by more than 10%. We checked the collinearity of the confounders using the variance inflation factor (VIF). The parsimony of the models was confirmed by Akaike information criterion.

We also performed two secondary analyses. Firstly, we tested if there was any effect modification by obesity on the association between AR + asthma, and the HRQoL and control scores. Secondly, we performed meta‐analyses to determine if there were any heterogeneity in the HRQoL and control (total) scores between the participating countries. All analyses were conducted using a complete case approach in Stata V.16 (StataCorp, College Station, TX, USA), and a *p*‐value <0.05 was considered statistically significant.

## 3 RESULTS

**FIGURE 1:**
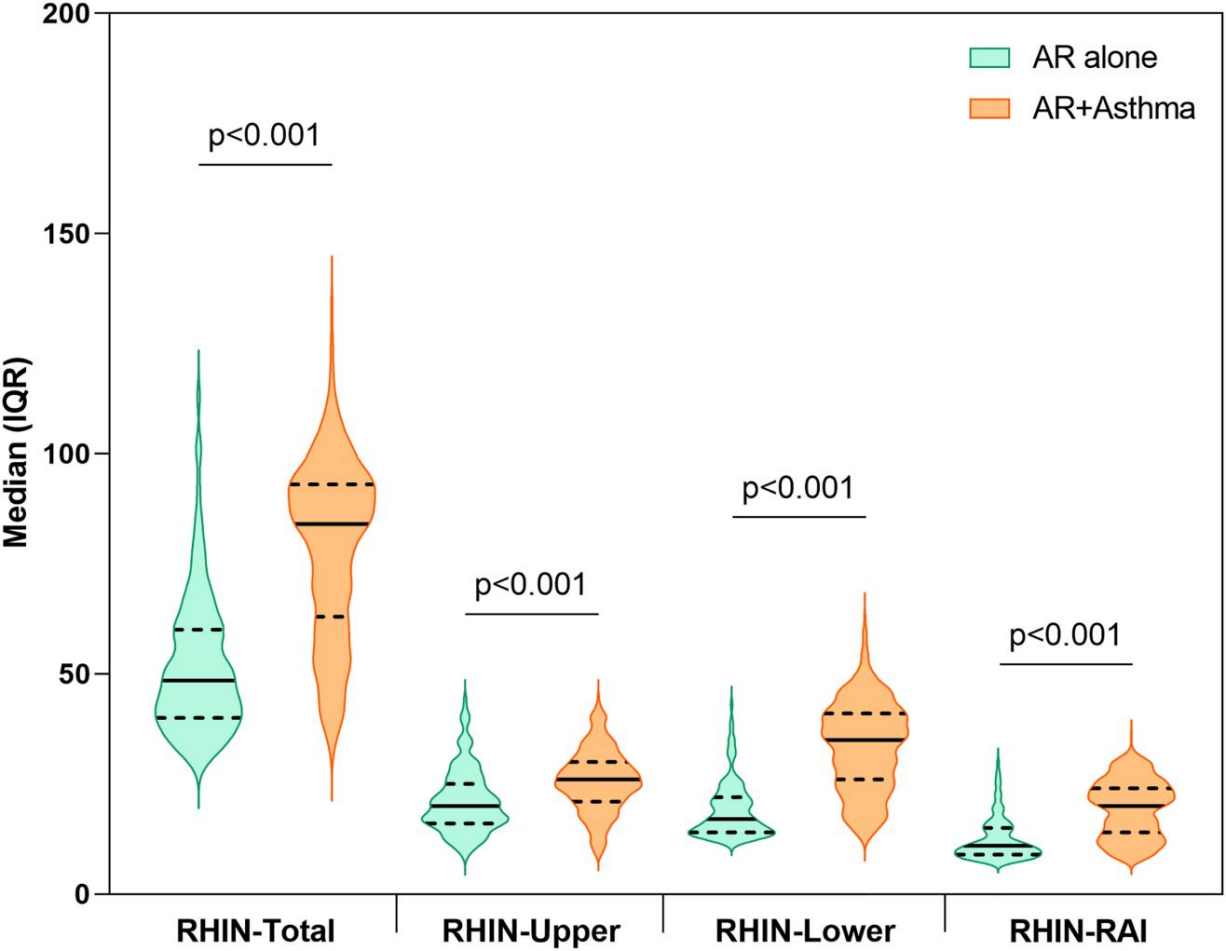
Differences in RHINASTHMA‐Total and subdomain scores between patients with Allergic rhinitis (AR) alone and AR + asthma. Data presented as median (solid line) and interquartile range (IQR) (dashed line) unless otherwise stated. *P*‐values were calculated from the Wilcoxon‐ranked sum test

**FIGURE 2:**
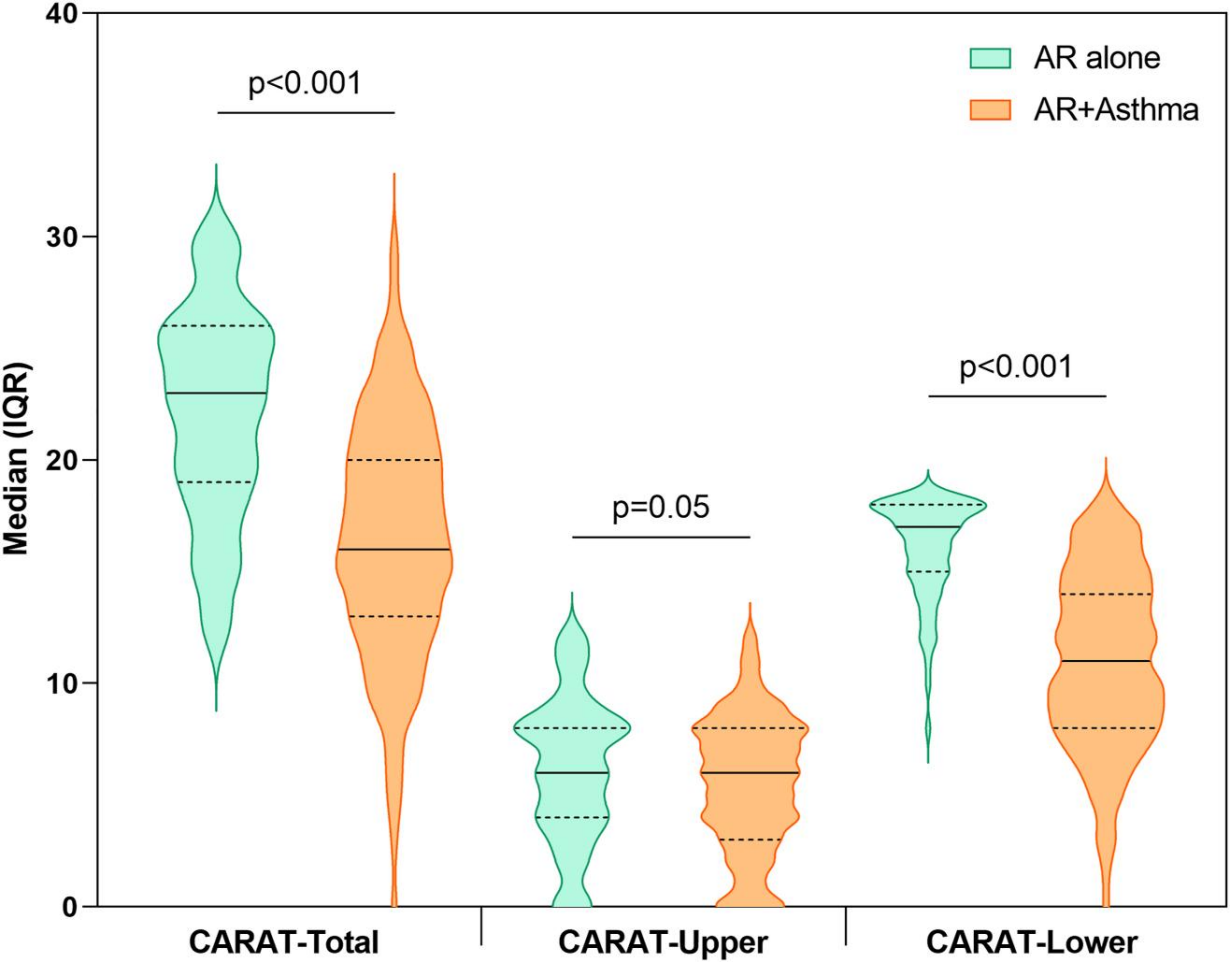
Differences in CARAT‐Total and subdomain scores between patients with Allergic rhinitis (AR) alone and AR + asthma. Data presented as median (solid line) and interquartile range (IQR) (dashed line) unless otherwise stated. *P*‐values were calculated from the Wilcoxon‐ranked sum test

The demographic and clinical characteristics of all the participants, stratified by country, are presented in Table 1. Of all participants, nearly 40% were males with a mean age of 44 (standard deviation, SD: 14) years, 15% of the participants were obese, 47% were smokers and nearly 33% reported exposure to smoke, 78% of the participants had asthma as comorbidity, 54% had moderate‐severe intermittent AR and 34% had moderate‐severe persistent AR. As for allergic sensitization, pollens were the most prevalent allergen (89%) among the participants, followed by house dust mites (57%). Concerning the HRQoL parameters, the participants had a median (IQR) RHIN‐Total score of 76 (53, 91) and CARAT‐Total score of 18 (14, 22). In the bivariate analysis, we found that participants with both AR and asthma had significantly higher RHINASTHMA (Total and subdomain) scores than the participants with AR alone (Figure 1). Moreover, CARAT (Total and subdomain) scores were significantly lower in AR with comorbid asthma than in AR alone (Figure 2).

**TABLE 1:** Descriptive statistics of the study patients overall and by country

	All (*N* = 643)	IT (*N* = 245)	FR (*N* = 212)	AT (*N* = 186)
Sex (male), *n* (%)	249 (38.9)	89 (36.3)	104 (49.5)	56 (30.1)
Age (years), mean (SD)	44.1 (14.4)	47.4 (14.2)	41.6 (15.3)	42.3 (12.5)
BMI, *n* (%)				
Underweight	31 (4.9)	6 (2.5)	21 (10.1)	4 (2.2)
Normal weight	358 (56.4)	144 (59.0)	118 (56.5)	96 (52.8)
Overweight	154 (24.3)	61 (25.0)	40 (19.1)	53 (29.1)
Obese	92 (14.5)	33 (13.5)	30 (14.4)	29 (15.9)
Smokers, *n* (%)	302 (47)	103 (42)	104 (49)	95 (51)
Exposure to smoke, *n* (%)	209 (32.6)	63 (25.8)	97 (45.8)	49 (26.3)
Education, *n* (%)				
Maximum 8 years	166 (25.9)	56 (22.9)	69 (32.9)	41 (22.0)
9–13 years	231 (36.0)	115 (46.9)	67 (31.9)	49 (26.3)
>13 years	244 (38.1)	74 (30.2)	74 (35.2)	96 (51.6)
GINA grade, *n* (%)				
No asthma	141 (22.0)	88 (35.9)	3 (1.4)	50 (26.9)
Intermittent	190 (29.6)	63 (25.7)	58 (27.6)	69 (37.1)
Mild persistent	36 (5.6)	13 (5.3)	13 (6.2)	10 (5.4)
Moderate persistent	60 (9.4)	20 (8.2)	27 (12.9)	13 (7.0)
Severe persistent	214 (33.4)	61 (24.9)	109 (51.9)	44 (23.7)
ARIA grade, *n* (%)				
Mild intermittent	60 (9.7)	40 (17.3)	7 (3.3)	13 (7.3)
Mild persistent	11 (1.8)	7 (3.0)	1 (0.5)	3 (1.7)
Moderate‐severe intermittent	336 (54.3)	111 (48.1)	149 (71.0)	76 (42.7)
Moderate‐severe persistent	212 (34.3)	73 (31.6)	53 (25.2)	86 (48.3)
Sensitivity to allergens, *n* (%)				
Sensitivity to at least one allergen	579 (90)	199 (81.1)	212 (100)	168 (90.3)
House dust mite	357 (56.9)	152 (64.1)	112 (53.1)	93 (51.7)
Moulds	123 (19.6)	32 (13.5)	51 (24.2)	40 (22.2)
Pollen	561 (89.2)	186 (78.5)	212 (100.0)	163 (90.6)
Dog	143 (22.8)	68 (28.7)	21 (10.0)	54 (30.0)
Cat	284 (45.2)	90 (38.0)	102 (48.3)	92 (51.1)
Birch	225 (35.8)	39 (16.5)	50 (23.7)	136 (75.6)
Cypress	110 (17.5)	38 (16.0)	57 (27.0)	15 (8.3)
Grass	275 (43.8)	149 (62.9)	53 (25.1)	73 (40.6)
Artemisia	82 (13.1)	6 (2.5)	47 (22.3)	29 (16.1)
Olive tree	173 (27.6)	78 (32.9)	57 (27.0)	38 (21.1)
*Parietaria* sp.	127 (20.2)	63 (26.6)	56 (26.5)	8 (4.4)
*Platanus* sp.	100 (15.9)	20 (8.4)	60 (28.4)	20 (11.1)
*Ambrosia* sp.	87 (13.9)	8 (3.4)	20 (9.5)	59 (32.8)
Sensitivity to at least one indoor allergen	469 (72.9)	167 (68.2)	171 (80.7)	131 (70.4)
Sensitivity to at least one outdoor allergen	561 (87.3)	186 (75.9)	212 (100.0)	163 (87.6)
Drugs taken in the last 12 months, *n* (%)	464 (72.2)	190 (77.6)	100 (47.2)	174 (93.6)
RHINASTHMA score, median (IQR)				
RHIN‐total (30 not at all–150 very much)	76 (53, 91)	52 (43, 67)	91 (85, 95)	70 (59, 91)
RHIN‐upper (9 not at all–45 very much)	25 (20, 29)	20 (16, 25)	27 (24, 30)	27 (21, 34)
RHIN‐lower (13 not at all–65 very much)	30 (21, 39)	20 (16, 26)	39 (36, 43)	28 (22, 36)
RHIN‐RAI (8 not at all–40 very much)	17 (12, 24)	12 (10, 15)	24 (22, 27)	16 (12, 21)
CARAT score, median (IQR)				
CARAT‐total (0 worst–30 best)	18 (14, 22)	22 (18, 25)	15 (13, 18)	16 (12, 20)
CARAT‐upper (0 worst–12 best)	6 (4, 8)	7 (4, 8)	6 (5, 8)	4 (1, 6)
CARAT‐lower (0 worst–18 best)	12 (9, 16)	16 (13, 17)	9 (7, 11)	12 (9, 15)

*Note*: Data presented as mean (SD) for continuous variables, median (interquartile range [IQR]) for ordinal variables, and frequency (%) for categorical variables.

Abbreviations: ARIA, Allergic Rhinitis Impact on Asthma guidelines; AT, Austria; BMI, Body Mass Index; CARAT, Control of Allergic Rhinitis and Asthma Test; FR, France; GINA, Global Initiative for Asthma; IT, Italy.

**FIGURE 3:**
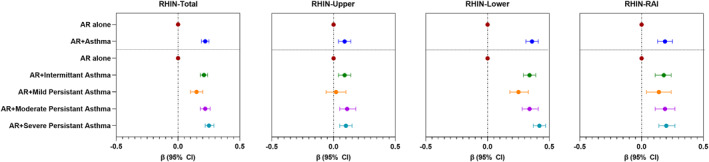
Adjusted association between AR + asthma and RHINASTHMA‐Total and subdomain scores. Data presented as regression coefficient (*β*) (symbol) and 95% confidence interval (CI) (horizontal bar) unless otherwise stated. Models were adjusted for age, sex, body mass index (BMI), smoking status, exposure to smoke, education, Allergic Rhinitis and its Impact on Asthma (ARIA) grade, sensitivity to allergens, and drugs taken in the last 12 months as fixed factors, and the country as a random factor

In the multivariable analysis, we observed that, compared to AR alone, AR with comorbid asthma was significantly associated with poorer QoL (regression coefficient [β] for RHIN‐Total score: 0.22; 95% confidence interval [CI]: 0.19, 0.25). The association was persistent in RHINASTHMA subdomain scores; however, the magnitude of the estimates was different, being the highest for lower airways (0.36; 0.31, 0.41) and the lowest for upper airways (0.09; 0.04, 0.14). Upon stratifying asthma, based on the 2017 GINA grades, the magnitude of the association was the highest in AR patients with severe persistent asthma (β for RHIN‐Total score: 0.25; 95%CI: 0.22, 0.29), and the lowest in AR patients with mild persistent asthma (0.15; 0.10, 0.20) (Figure 3 and Supplementary Table 1). We did not find any multicollinearity between the covariates (VIF<3).

**FIGURE 4:**
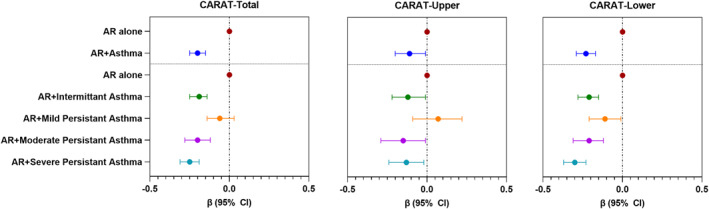
Adjusted association between AR + asthma and CARAT‐Total and subdomain scores. Data presented as regression coefficient (*β*) (symbol) and 95% confidence interval (CI) (horizontal bar) unless otherwise stated. Models were adjusted for age, sex, body mass index (BMI), smoking status, exposure to smoke, education, Allergic Rhinitis and its Impact on Asthma (ARIA) grade, sensitivity to allergens, and drugs taken in the last 12 months as fixed factors, and the country as a random factor

We observed a poorer control of symptoms in AR patients with asthma comorbidity than in patients with AR alone (β for CARAT‐Total score: −0.20; 95%CI: −0.25, −0.15); the lower airway symptoms were more poorly controlled (−0.23; −0.29, −0.17) than the upper airway symptoms (−0.11; −0.20, −0.01). Upon stratifying asthma according to GINA grade, AR patients with severe persistent asthma had the poorest control (β for CARAT‐Total score: −0.25; 95%CI: −0.31, −0.19) than those with mild persistent asthma (−0.06; −0.14, 0.03) (Figure 4 and Supplementary Table 2).

In the sensitivity analysis for effect modification by obesity (Supplementary Table 3), we found that the association between AR + asthma and RHIN‐Total score was marginally higher among non‐obese participants (β: 0.23; 95%CI: 0.19, 0.26) than obese ones (0.16; 0.07, 0.25) (*p‐value* for interaction = 0.09). The difference was more pronounced for upper airways, the association being significantly higher among non‐obese participants (β for RHIN‐Upper: 0.10; 0.05, 0.15) than obese ones (0.01; −0.13, 0.15) (*p‐value* for interaction = 0.04). However, we did not observe significant differences in other subdomains. We did not observe any effect modification by obesity on CARAT scores (Supplementary Table 4).

**FIGURE 5:**
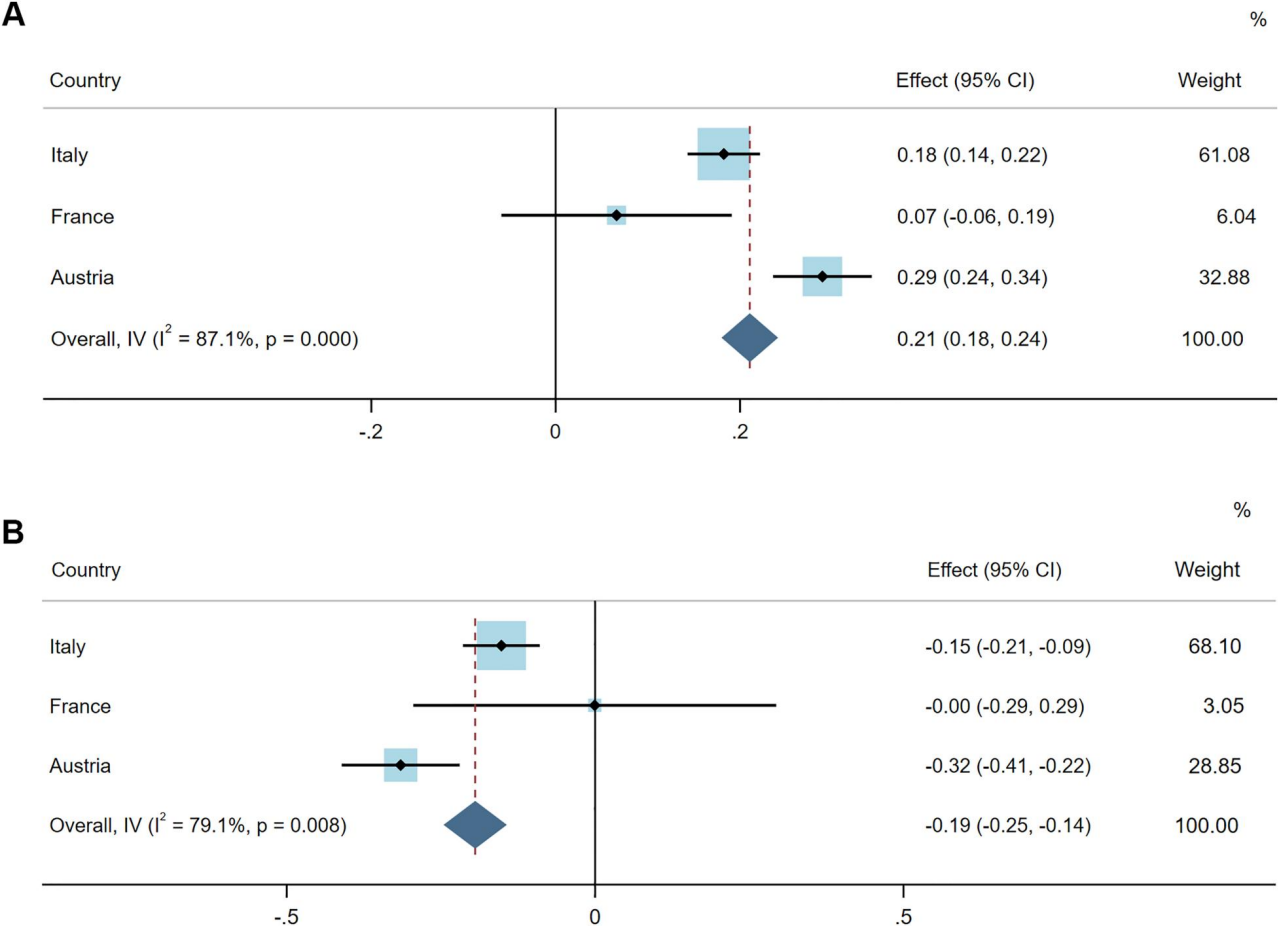
Meta‐analysis results of the association between AR + asthma and (A) RHIN‐Total score and (B) CARAT‐Total score, stratified by countries. Models were adjusted for sex, age, smoking status, exposure to smoke, education, Allergic Rhinitis and its Impact on Asthma (ARIA) grade, sensitivity to allergens, and drugs taken in the last 12 months as fixed factors. I‐squared, variation in estimated effect attributable to heterogeneity

The association of AR + asthma with RHIN‐Total score was highly heterogeneous (I^2^: 87%; *p*‐value for heterogeneity = <0.001) across the participating countries (Figure 5A). While the association was the highest in Austria (β: 0.29; 95%CI: 0.24, 0.34), it tended toward null in France (0.07, −0.06, 0.19). Similar heterogeneity was observed for CARAT‐Total score (I^2^: 79%, *p* = 0.008) (Figure 5B). However, the overall estimates from the meta‐analyses for the association between AR + asthma, and RHIN‐Total and CARAT‐Total scores were similar to the ones reported in the main analysis.

## 4 DISCUSSION

In our study, we found a significantly worse QoL (RHINASTHMA Total and subdomain scores) and symptoms control (CARAT Total and subdomain scores) in patients with AR + asthma than in patients with AR alone. We also found that the association was significantly higher among non‐obese participants compared to obese ones, when assessed through RHIN‐Upper symptoms score but not with CARAT. We also observed country‐specific variations in the RHINASTHMA and CARAT Total scores. Although one previous study compared the individual/social burden of disease between asthmatics and asthmatics with concomitant AR, unlike ours, that study did not compare the difference in disease control and HRQoL between the two groups of patients.^25^

It is well‐known that several triggers such as seasonal meteorological changes, pollen season, air pollution, or even occupational exposures may lead to poor QoL of asthmatic patients with or without AR.^8^, ^26^, ^27^, ^28^ It has also been observed that AR patients are often reported to have poor control over their symptoms if persistent comorbid asthma is present.^29^, ^30^, ^31^, ^32^ Although no direct comparative study on the control and HRQoL of AR and AR with asthma has been reported yet, our findings well reciprocate the previous results. Asthma and AR share eight common genes (*CLC, EMR4P, IL5RA, FRRS1, HRH4, SLC29A1, SIGLEC8, IL1RL1*) that are presumed to describe the link for multimorbidity.^33^ They also share common risk factors such as atopic genetic background (for the allergic endotypes), environmental exposures (allergens, moulds, indoor and outdoor air pollution, some respiratory viruses, etc.), type of occupation, and active tobacco smoking.

We found that the non‐obese patients with AR + asthma had poorer QoL (RHIN‐Upper score) than their obese counterparts. Reports citing the role of higher BMI on the QoL in patients with an upper airway disease have been mixed. While some studies reported poorer asthma QoL in asthmatics with higher BMI,^34^, ^35^ other studies did not observe any significant role of higher BMI on QoL in allergic/asthma patients.^36^ Our result could also be influenced by other factors such as physical activity or environmental conditions; however, we could not confirm them in our study.

We found significant country‐wise heterogeneity in RHINASTHMA and CARAT‐Total scores between AR alone and AR + asthma. This could be explained by a higher number of AR + asthma patients in France than in the two other countries. Another reason for such heterogeneity could be due to significant variation in allergen sensitivity between the countries, which has also been previously reported describing a significant difference in aeroallergens and allergies between European countries.^37^, ^38^, ^39^ Air pollution is another important perturbation that can significantly affect HRQoL and symptom control in allergic patients and a recent meta‐analysis suggested that there is significant variability in air pollution between different European counties that have differently attributed to the risk of AR.^40^ However, studying air pollution was beyond the scope of our current study. Nevertheless, this variation of HRQoL and control could also be influenced by other, such as co‐occurrence of any food allergies, and other environmental conditions, which we could not assess in this current study.

Our findings add important clinical knowledge to the existing strategies for the management of AR with concomitant asthma. Although AR and asthma are two different diseases with distinct clinical features, when AR persists with asthma, either condition is often overlooked^32^, ^41^ due to the lack of a combined tool for monitoring control and HRQoL of both diseases at the same time. Despite the well‐established guidelines of ARIA and GARD for a new management protocol for AR and asthma together,^10^, ^12^, ^42^, ^43^, ^44^, ^45^ reports adopting these guidelines in the management of AR with persistent asthma are still lacking. Our findings would help guide practitioners to use the appropriate assessment tools while treating such patients. Our study recruited patients from three European countries which have distinct geographical, climatic, and aerobiological conditions. Moreover, we recorded sensitivity data to a wide variety of indoor and outdoor allergens which enabled us to observe the distribution of those allergens across the participant countries. Our meta‐analytic approach to assessing country‐wise variation in HRQoL and control provides a novel understanding of the divergent population and their disease conditions. Our findings underline the impact of respiratory hypersensitivity conditions on the QoL of patients and call for prevention and public health strategies to diminish the burden of these conditions. Currently, there are effective treatments for AR and asthma, several risk factors are known (*e.g.,* allergies, rhinitis, tobacco smoke) and tools to control the disease have been developed. However, we are still uncertain how to prevent AR patients from developing asthma, allergen immunotherapy being the current only attempt. Preventive measures should be able to change the natural history of the disorder, avoiding asthma development in patients with AR and/or evolution by providing its control.^46^

Our study has some limitations. Firstly, considering that subjective symptom‐rating scales may not be entirely accurate, the risk for potential bias could not be completely avoided. However, we used standardized instruments, and therefore the possibility of such bias was marginal. Secondly, the considered period might be insufficient to evaluate the QoL and the control appropriately. Thirdly, other comorbidities might have modified the patients' responses. Despite these limitations, our findings are derived from incident patients drawn from the general population of three European countries in which AR and asthma diagnoses were made by a doctor. However, due to the small sample size, it is not possible to indicate whether these results may be generalized. Further studies, after controlling for potential confounders and biases in larger populations, are therefore warranted.

## 5 CONCLUSION

In summary, using combined assessment tools for AR and asthma, we found that AR patients with comorbid asthma have a poorer quality of life and symptom control than those with AR alone. This finding highlights the importance of a comprehensive approach for the management of AR in case of a comorbid allergic condition for optimum care, and such strategies would be the gateway to reducing the global burden of these diseases.

## AUTHOR CONTRIBUTIONS

**Subhabrata Moitra**: Formal analysis (Lead); Software (Lead); Writing – original draft (Lead), Writing – review & editing (Equal). **Marzia Simoni**: Conceptualization (Equal); Data curation (Equal); Funding acquisition (Equal); Investigation (Equal); Methodology (Equal); Project administration (Equal); Resources (Equal); Supervision (Equal); Validation (Equal); Visualization (Equal); Writing – review & editing (Equal). **Sandra Baldacci**: Conceptualization (Equal); Data curation (Equal); Funding acquisition (Equal); Investigation (Equal); Methodology (Equal); Project administration (Equal); Resources (Equal); Supervision (Equal); Validation (Equal); Visualization (Equal); Writing – review & editing (Equal). **Sara Maio**: Conceptualization (Equal); Data curation (Equal); Funding acquisition (Equal); Investigation (Equal); Methodology (Equal); Project administration (Equal); Resources (Equal); Supervision (Equal); Validation (Equal); Visualization (Equal); Writing – review & editing (Equal). **Anna Angino**: Conceptualization (Equal); Data curation (Equal); Funding acquisition (Equal); Investigation (Equal); Methodology (Equal); Project administration (Equal); Resources (Equal); Supervision (Equal); Validation (Equal); Visualization (Equal); Writing – review & editing (Equal). **Patrizia Silvi**: Conceptualization (Equal); Data curation (Equal); Funding acquisition (Equal); Investigation (Equal); Methodology (Equal); Project administration (Equal); Resources (Equal); Supervision (Equal); Validation (Equal); Visualization (Equal); Writing – review & editing (Equal). **Giovanni Viegi**: Conceptualization (Equal); Data curation (Equal); Funding acquisition (Equal); Investigation (Equal); Methodology (Equal); Project administration (Equal); Resources (Equal); Supervision (Equal); Validation (Equal); Visualization (Equal); Writing – review & editing (Equal). **Stefania La Grutta**: Conceptualization (Equal); Data curation (Equal); Funding acquisition (Equal); Investigation (Equal); Methodology (Equal); Project administration (Equal); Resources (Equal); Supervision (Equal); Validation (Equal); Visualization (Equal); Writing – review & editing (Equal). **Franco Ruggiero**: Conceptualization (Equal); Data curation (Equal); Funding acquisition (Equal); Investigation (Equal); Methodology (Equal); Project administration (Equal); Resources (Equal); Supervision (Equal); Validation (Equal); Visualization (Equal); Writing – review & editing (Equal). **Gianni Bedini**: Conceptualization (Equal); Data curation (Equal); Funding acquisition (Equal); Investigation (Equal); Methodology (Equal); Project administration (Equal); Resources (Equal); Supervision (Equal); Validation (Equal); Visualization (Equal); Writing – review & editing (Equal). **Francesca Natali**: Conceptualization (Equal); Data curation (Equal); Funding acquisition (Equal); Investigation (Equal); Methodology (Equal); Project administration (Equal); Resources (Equal); Supervision (Equal); Validation (Equal); Visualization (Equal); Writing – review & editing (Equal). **Lorenzo Cecchi**: Conceptualization (Equal); Data curation (Equal); Funding acquisition (Equal); Investigation (Equal); Methodology (Equal); Project administration (Equal); Resources (Equal); Supervision (Equal); Validation (Equal); Visualization (Equal); Writing – review & editing (Equal). **Uwe Berger**: Conceptualization (Equal); Data curation (Equal); Funding acquisition (Equal); Investigation (Equal); Methodology (Equal); Project administration (Equal); Resources (Equal); Supervision (Equal); Validation (Equal); Visualization (Equal); Writing – review & editing (Equal). **Maria Prentovic**: Conceptualization (Equal); Data curation (Equal); Funding acquisition (Equal); Investigation (Equal); Methodology (Equal); Project administration (Equal); Resources (Equal); Supervision (Equal); Validation (Equal); Visualization (Equal); Writing – review & editing (Equal). **Amir Gamil**: Conceptualization (Equal); Data curation (Equal); Funding acquisition (Equal); Investigation (Equal); Methodology (Equal); Project administration (Equal); Resources (Equal); Supervision (Equal); Validation (Equal); Visualization (Equal); Writing – review & editing (Equal). **Nour Baiz**: Conceptualization (Equal); Data curation (Equal); Funding acquisition (Equal); Investigation (Equal); Methodology (Equal); Project administration (Equal); Resources (Equal); Supervision (Equal); Validation (Equal); Visualization (Equal); Writing – review & editing (Equal). **Michel Thibaudon**: Conceptualization (Equal); Data curation (Equal); Funding acquisition (Equal); Investigation (Equal); Methodology (Equal); Project administration (Equal); Resources (Equal); Supervision (Equal); Validation (Equal); Visualization (Equal); Writing – review & editing (Equal). **Samuel Monnier**: Conceptualization (Equal); Data curation (Equal); Funding acquisition (Equal); Investigation (Equal); Methodology (Equal); Project administration (Equal); Resources (Equal); Supervision (Equal); Validation (Equal); Visualization (Equal); Writing–review & editing (Equal). **Davide Caimmi**: Conceptualization (Equal); Data curation (Equal); Funding acquisition (Equal); Investigation (Equal); Methodology (Equal); Project administration (Equal); Resources (Equal); Supervision (Equal); Validation (Equal); Visualization (Equal); Writing – review & editing (Equal). **Luciana K. Tanno**: Conceptualization (Equal); Data curation (Equal); Funding acquisition (Equal); Investigation (Equal); Methodology (Equal); Project administration (Equal); Resources (Equal); Supervision (Equal); Validation (Equal); Visualization (Equal); Writing – review & editing (Equal). **Pascal Demoly**: Conceptualization (Equal); Data curation (Equal); Funding acquisition (Equal); Investigation (Equal); Methodology (Equal); Project administration (Equal); Resources (Equal); Supervision (Equal); Validation (Equal); Visualization (Equal); Writing – review & editing (Equal). **Simone Orlandini**: Conceptualization (Equal); Data curation (Equal); Funding acquisition (Equal); Investigation (Equal); Methodology (Equal); Project administration (Equal); Resources (Equal); Supervision (Equal); Validation (Equal); Visualization (Equal); Writing–review & editing (Equal). **Isabella Annesi‐Maesano**: Conceptualization (Lead); Data curation (Lead); Funding acquisition (Equal); Investigation (Equal); Methodology (Equal); Project administration (Equal); Resources (Lead); Supervision (Lead); Validation (Equal); Visualization (Equal); Writing – review & editing (Equal).

## CONFLICT OF INTEREST

Subhabrata Moitra reports personal fees from Synergy Respiratory & Cardiac Care (Canada), Permanyer Inc. (Spain), Elsevier Inc., and Apollo Gleneagles Hospital (India) outside this submitted work. Uwe Berger reports grants from CAMS‐32 outside the submitted work. Pascal Demoly received grants from ALK, Stallergenes Greer, AstraZeneca, ThermoFisherScientific, Ménarini, GlaxoSmithKline, Zambon, Viatris, and personal fees from Chiesi, and Puuressentiel, outside the submitted work. Pascal Demoly is also the Vice President of the French Allergy Society and Immediate Past President of the French Allergy Council. Isabella Annesi‐Maesano reports grants from the European Council and other public bodies outside the submitted work. Isabella Annesi‐Maesano is also the President of the Institut Recherche et Dévelopment Ethics Committee and a Member of the European Respiratory Society Ethics and Integrity Committee. Other authors do not have any conflict of interest to declare.

## Supporting information

Supplementary Material 1
