# Supplementary material for: Symptom control and health‐related quality of life in allergic rhinitis with and without comorbid asthma: A multicentre European study
Source: Clin Transl Allergy. 2023 Feb 2;13(2):e12209. doi: 10.1002/clt2.12209 (PMC9893887; doi:10.1002/clt2.12209)
Supplement: Supplementary file 1 — Supplementary Material 1 [file CLT2-13-e12209-s001.docx]

SUPPLEMENT

**Symptom control and health-related quality of life in allergic rhinitis with and without comorbid asthma: a multicentre European study**

Subhabrata Moitra, Marzia Simoni, Sandra Baldacci, Sara Maio, Anna Angino, Patrizia Silvi, Giovanni Viegi, Stefania La Grutta, Franco Ruggiero, Gianni Bedini, Francesca Natali, Lorenzo Cecchi, Uwe Berger, Maria Prentovic, Amir Gamil, Nour Baïz, Michel Thibaudon, Samuel Monnier, Davide Caimmi, Luciana K. Tanno, Pascal Demoly, Simone Orlandini, Isabella Annesi-Maesano

**List of Supplementary Tables**

**Supplementary Table 1:** Association between AR+asthma and RHINASTHMA score

**Supplementary Table 2:** Association between AR+asthma and CARAT score

**Supplementary Table 3:** Effect modification of the association between AR+asthma and RHINASTHMA-total score by obesity.

**Supplementary Table 4:** Effect modification of the association between AR+asthma and CARAT-total score by obesity.

**Supplementary Table 1:** Association between AR+asthma and RHINASTHMA score

|  | **RHIN – Total** | | **RHIN – Upper** | | **RHIN – Lower** | | **RHIN – RAI** | |
| --- | --- | --- | --- | --- | --- | --- | --- | --- |
|  | **β (95%CI)** | | **β (95%CI)** | | **β (95%CI)** | | **β (95%CI)** | |
|  | **Unadjusted** | **Adjusted** | **Unadjusted** | **Adjusted** | **Unadjusted** | **Adjusted** | **Unadjusted** | **Adjusted** |
| AR alone | Ref | Ref | Ref | Ref | Ref | Ref | Ref | Ref |
| AR+Asthma | **0.26 (0.24, 0.29)** | **0.22 (0.19, 0.25)** | **0.14 (0.09, 0.18)** | **0.09 (0.04, 0.14)** | **0.41 (0.36, 0.45)** | **0.36 (0.31, 0.41)** | **0.24 (0.18, 0.29)** | **0.19 (0.13, 0.25)** |
| AR alone | Ref | Ref | Ref | Ref | Ref | Ref | Ref | Ref |
| AR+Intermittent asthma | **0.24 (0.21, 0.27)** | **0.21 (0.18, 0.24)** | **0.13 (0.08, 0.17)** | **0.09 (0.04, 0.14)** | **0.38 (0.33, 0.42)** | **0.34 (0.29, 0.39)** | **0.22 (0.16, 0.28)** | **0.18 (0.11, 0.24)** |
| AR+Mild persistent asthma | **0.18 (0.13, 0.23)** | **0.15 (0.10, 0.20)** | 0.06 (-0.02, 0.14) | 0.02 (-0.06, 0.10) | **0.28 (0.21, 0.35)** | **0.25 (0.18, 0.33)** | **0.17 (0.08, 0.27)** | **0.14 (0.04, 0.24)** |
| AR+Moderate persistent asthma | **0.26 (0.22, 0.30)** | **0.22 (0.18, 0.26)** | **0.17 (0.10, 0.23)** | **0.11 (0.05, 0.18)** | **0.40 (0.33, 0.46)** | **0.34 (0.28, 0.41)** | **0.23 (0.16, 0.31)** | **0.19 (0.11, 0.27)** |
| AR+Severe persistent asthma | **0.31 (0.28, 0.34)** | **0.25 (0.22, 0.29)** | **0.15 (0.10, 0.20)** | **0.10 (0.05, 0.15)** | **0.47 (0.42, 0.52)** | **0.42 (0.37, 0.47)** | **0.27 (0.21, 0.33)** | **0.20 (0.14, 0.27)** |

Data presented as regression coefficient (β) and 95% confidence interval (95%CI) from mixed-effect negative binomial regression. Two sets of regression models were created – i) no asthma vs. asthma and ii) no asthma vs. asthma phenotypes. The models were adjusted for sex, age, smoking status, exposure to smoke, education, ARIA grade, and drugs taken in the last 12 months as fixed factors, and the country as a random factor. Abbreviations: RHIN: RHINASTHMA score; RHIN-RAI: RHINASTHMA score – respiratory allergy impact

**Supplementary Table 2:** Association between AR+asthma and CARAT score

|  | **CARAT – Total** | | **CARAT – Upper** | | **CARAT – Lower** | |
| --- | --- | --- | --- | --- | --- | --- |
|  | **β (95%CI)** | | **β (95%CI)** | | **β (95%CI)** | |
|  | **Unadjusted** | **Adjusted** | **Unadjusted** | **Adjusted** | **Unadjusted** | **Adjusted** |
| AR alone | Ref | Ref | Ref | Ref | Ref | Ref |
| AR+Asthma | **-0.23 (-0.28, -0.19)** | **-0.20 (-0.25, -0.15)** | **-0.17 (-0.25, -0.08)** | **-0.11 (-0.20, -0.01)** | **-0.26 (-0.32, -0.21)** | **-0.23 (-0.29, -0.17)** |
| AR alone | Ref | Ref | Ref | Ref | Ref | Ref |
| AR+Intermittent asthma | **-0.21 (-0.26, -0.16)** | **-0.19 (-0.25, -0.14)** | **-0.16 (-0.26, -0.06)** | **-0.12 (-0.22, -0.01)** | **-0.23 (-0.29, -0.17)** | **-0.21 (-0.28, -0.15)** |
| AR+Mild persistent asthma | -0.07 (-0.16, 0.01) | -0.06 (-0.14, 0.03) | 0.01 (-0.14, 0.16) | 0.07 (-0.09, 0.22) | **-0.12 (-0.22, -0.02)** | **-0.11 (-0.21, -0.01)** |
| AR+Moderate persistent asthma | **-0.24 (-0.31, -0.16)** | **-0.20 (-0.28, -0.12)** | **-0.20 (-0.33, -0.07)** | **-0.15 (-0.29, -0.01)** | **-0.25 (-0.34, -0.16)** | **-0.21 (-0.31, -0.12)** |
| AR+Severe persistent asthma | **-0.30 (-0.35, -0.24)** | **-0.25 (-0.31, -0.19)** | **-0.21 (-0.30, -0.11)** | **-0.13 (-0.24, -0.02)** | **-0.35 (-0.41, -0.28)** | **-0.30 (-0.37, -0.23)** |

Data presented as regression coefficient (β) and 95% confidence interval (95%CI). Two sets of regression models were created – i) no asthma vs. asthma and ii) no asthma vs. asthma phenotypes. The models were adjusted for sex, age, smoking status, exposure to smoke, education, ARIA grade, and drugs taken in the last 12 months as fixed factors, and the country as a random factor. Abbreviations: CARAT: Control of asthma and allergic rhinitis test

**Supplementary Table 3:** Effect modification of the association between AR+asthma and RHINASTHMA score by obesity

|  | **RHIN – Total** | | | **RHIN – Upper** | | | **RHIN – Lower** | | | **RHIN – RAI** | | |
| --- | --- | --- | --- | --- | --- | --- | --- | --- | --- | --- | --- | --- |
|  | **β (95%CI)** | | **^#^P** | **β (95%CI)** | | **^#^P** | **β (95%CI)** | | **^#^P** | **β (95%CI)** | | **^#^P** |
|  | **Obese** | |  | **Obese** | |  | **Obese** | |  | **Obese** | |  |
|  | **No (n=543)** | **Yes (n=92)** |  | **No (n=543)** | **Yes (n=92)** |  | **No (n=543)** | **Yes (n=92)** |  | **No** | **No (n=543)** |  |
| AR alone | Ref | Ref | 0.09 | Ref | Ref | **0.04** | Ref | Ref | 0.55 | Ref | Ref | 0.57 |
| AR+Asthma | **0.23 (0.19, 0.26)** | **0.16 (0.07, 0.25)** |  | **0.10 (0.05, 0.15)** | 0.01 (-0.13, 0.15) |  | **0.35 (0.30, 0.40)** | **0.35 (0.20, 0.50)** |  | **0.18 (0.11, 0.24)** | **0.21 (0.02, 0.40)** |  |
| AR alone | Ref | Ref | 0.07 | Ref | Ref | 0.28 | Ref | Ref | 0.50 | Ref | Ref | 0.24 |
| AR+Intermittent asthma | **0.22 (0.18, 0.25)** | **0.16 (0.06, 0.26)** |  | **0.10 (0.05, 0.16)** | 0.02 (-0.14, 0.17) |  | **0.34 (0.29, 0.40)** | **0.34 (0.18, 0.50)** |  | **0.17 (0.11, 0.24)** | **0.22 (0.01, 0.43)** |  |
| AR+Mild persistent asthma | **0.15 (0.10, 0.21)** | 0.10 (-0.02, 0.22) |  | 0.05 (-0.04, 0.15) | -0.06 (-0.26, 0.13) |  | **0.23 (0.14, 0.32)** | **0.30 (0.10, 0.49)** |  | **0.12 (0.01, 0.23)** | 0.17 (-0.07, 0.41) |  |
| AR+Moderate persistent asthma | **0.23 (0.19, 0.28)** | 0.08 (-0.04, 0.20) |  | **0.13 (0.06, 0.20)** | -0.04 (-0.23, 0.16) |  | **0.35 (0.28, 0.42)** | **0.24 (0.04, 0.43)** |  | **0.20 (0.11, 0.29)** | 0.08 (-0.18, 0.33) |  |
| AR+Severe persistent asthma | **0.25 (0.22, 0.29)** | **0.25 (0.15, 0.34)** |  | **0.11 (0.05, 0.17)** | 0.04 (-0.11, 0.19) |  | **0.40 (0.35, 0.46)** | **0.46 (0.30, 0.62)** |  | **0.19 (0.12, 0.26)** | 0.29 (0.09, 0.50) |  |

Data presented as regression coefficient (β) and 95% confidence interval (95%CI). Two sets of regression models were created – i) no asthma vs. asthma and ii) no asthma vs. asthma phenotypes. The models were adjusted for sex, age, smoking status, exposure to smoke, education, ARIA grade, and drugs taken in the last 12 months as fixed factors, and the country as a random factor. Obesity was considered if BMI > 30 kg/m^2^.

^#^p values for interaction

**Supplementary Table 4:** Effect modification of the association between AR+asthma and CARAT score by obesity

|  | **CARAT – Total** | | | **CARAT – Upper** | | | **CARAT – Lower** | | |
| --- | --- | --- | --- | --- | --- | --- | --- | --- | --- |
|  | **β (95%CI)** | | **^#^P** | **β (95%CI)** | | **^#^P** | **β (95%CI)** | | **^#^P** |
|  | **Obese** | |  | **Obese** | |  | **Obese** | |  |
|  | **No (n=543)** | **Yes (n=92)** |  | **No (n=543)** | **Yes (n=92)** |  | **No (n=543)** | **Yes (n=92)** |  |
| AR alone | Ref | Ref | 0.66 | Ref | Ref | 0.44 | Ref | Ref | 0.99 |
| AR+Asthma | **-0.20 (-0.26, -0.15)** | **-0.18 (-0.34, -0.01)** |  | -0.14 (-0.25, -0.04) | 0.08 (-0.21, 0.36) |  | **-0.22 (-0.29, -0.16)** | **-0.29 (-0.49, -0.10)** |  |
| AR alone | Ref | Ref | 0.62 | Ref | Ref | 0.88 | Ref | Ref | 0.37 |
| AR+Intermittent asthma | **-0.20 (-0.26, -0.14)** | -0.18 (-0.36, 0.0002) |  | -0.16 (-0.27, -0.04) | 0.08 (-0.24, 0.40) |  | **-0.21 (-0.28, -0.14)** | **-0.28 (-0.50, -0.06)** |  |
| AR+Mild persistent asthma | -0.05 (-0.15, 0.05) | -0.03 (-0.25, 0.20) |  | 0.04 (-0.14, 0.22) | 0.24 (-0.17, 0.65) |  | **-0.08 (-0.20, 0.04)** | -0.21 (-0.49, 0.07) |  |
| AR+Moderate persistent asthma | -0.22 (-0.31, -0.14) | -0.10 (-0.32, 0.13) |  | **-0.19 (-0.34, -0.03)** | -0.12 (-0.53, 0.30) |  | **-0.23 (-0.34, -0.13)** | -0.07 (-0.33, 0.19) |  |
| AR+Severe persistent asthma | **-0.25 (-0.31, -0.18)** | **-0.28 (-0.46, -0.09)** |  | **-0.17 (-0.28, -0.05)** | 0.10 (-0.22, 0.42) |  | **-0.28 (-0.36, -0.20)** | **-0.47 (-0.69, -0.26)** |  |

Data presented as regression coefficient (β) and 95% confidence interval (95%CI). Two sets of regression models were created – i) no asthma vs. asthma and ii) no asthma vs. asthma phenotypes. The models were adjusted for sex, age, smoking status, exposure to smoke, education, ARIA grade, and drugs taken in the last 12 months as fixed factors, and the country as a random factor. Obesity was considered if BMI > 30 kg/m^2^

^#^p values were obtained for interaction.
